# Systematic literature review of schizophrenia clinical practice guidelines on acute and maintenance management with antipsychotics

**DOI:** 10.1038/s41537-021-00192-x

**Published:** 2022-02-24

**Authors:** Christoph U. Correll, Amber Martin, Charmi Patel, Carmela Benson, Rebecca Goulding, Jennifer Kern-Sliwa, Kruti Joshi, Emma Schiller, Edward Kim

**Affiliations:** 1 The Zucker Hillside Hospital, Department of Psychiatry, Northwell Health, Glen Oaks, NY USA; 2 Donald and Barbara Zucker School of Medicine at Hofstra/Northwell, Department of Psychiatry and Molecular Medicine, Hempstead, NY USA; 3 Charité Universitätsmedizin Berlin, Department of Child and Adolescent Psychiatry, Berlin, Germany; 4 Evidera, Waltham, MA USA; 5 Janssen Scientific Affairs, LLC, Titusville, NJ USA; 6 Goulding HEOR Consulting Inc., Vancouver, BC Canada; 7 Biohaven Pharmaceuticals, New Haven, CT USA

**Keywords:** Schizophrenia, Psychology

## Abstract

Clinical practice guidelines (CPGs) translate evidence into recommendations to improve patient care and outcomes. To provide an overview of schizophrenia CPGs, we conducted a systematic literature review of English-language CPGs and synthesized current recommendations for the acute and maintenance management with antipsychotics. Searches for schizophrenia CPGs were conducted in MEDLINE/Embase from 1/1/2004–12/19/2019 and in guideline websites until 06/01/2020. Of 19 CPGs, 17 (89.5%) commented on first-episode schizophrenia (FES), with all recommending antipsychotic monotherapy, but without agreement on preferred antipsychotic. Of 18 CPGs commenting on maintenance therapy, 10 (55.6%) made no recommendations on the appropriate maximum duration of maintenance therapy, noting instead individualization of care. Eighteen (94.7%) CPGs commented on long-acting injectable antipsychotics (LAIs), mainly in cases of nonadherence (77.8%), maintenance care (72.2%), or patient preference (66.7%), with 5 (27.8%) CPGs recommending LAIs for FES. For treatment-resistant schizophrenia, 15/15 CPGs recommended clozapine. Only 7/19 (38.8%) CPGs included a treatment algorithm.

## Introduction

Schizophrenia is an often debilitating, chronic, and relapsing mental disorder with complex symptomology that manifests as a combination of positive, negative, and/or cognitive features^1–3^. Standard management of schizophrenia includes the use of antipsychotic medications to help control acute psychotic episodes^4^ and prevent relapses^5,6^, whereas maintenance therapy is used in the long term after patients have been stabilized^7–9^. Two main classes of drugs—first- and second-generation antipsychotics (FGA and SGA)—are used to treat schizophrenia^10^. SGAs are favored due to the lower rates of adverse effects, such as extrapyramidal effects, tardive dyskinesia, and relapse^11^. However, pharmacologic treatment for schizophrenia is complicated because nonadherence is prevalent, and is a major risk factor for relapse^9^ and poor overall outcomes^12^. The use of long-acting injectable (LAI) versions of antipsychotics aims to limit nonadherence-related relapses and poor outcomes^13^.

Patient treatment pathways and treatment choices are determined based on illness acuity/severity, past treatment response and tolerability, as well as balancing medication efficacy and adverse effect profiles in the context of patient preferences and adherence patterns^14,15^. Clinical practice guidelines (CPG) serve to inform clinicians with recommendations that reflect current evidence from meta-analyses of randomized controlled trials (RCTs), individual RCTs and, less so, epidemiologic studies, as well as clinical experience, with the goal of providing a framework and road-map for treatment decisions that will improve quality of care and achieve better patients outcomes. The use of clinical algorithms or other decision trees in CPGs may improve the ease of implementation of the evidence in clinical practice^16^. While CPGs are an important tool for mental health professionals, they have not been updated on a regular basis like they have been in other areas of medicine, such as in oncology. In the absence of current information, other governing bodies, healthcare systems, and hospitals have developed their own CPGs regarding the treatment of schizophrenia, and many of these have been recently updated^17–19^. As such, it is important to assess the latest guidelines to be aware of the changes resulting from consideration of updated evidence that informed the treatment recommendations. Since CPGs are comprehensive and include the diagnosis as well as the pharmacological and non-pharmacological management of individuals with schizophrenia, a detailed comparative review of all aspects of CPGs for schizophrenia would have been too broad a review topic. Further, despite ongoing efforts to broaden the pharmacologic tools for the treatment of schizophrenia^20^, antipsychotics remain the cornerstone of schizophrenia management^8,21^. Therefore, a focused review of guideline recommendations for the management of schizophrenia with antipsychotics would serve to provide clinicians with relevant information for treatment decisions.

To provide an updated overview of United States (US) national and English language international guidelines for the management of schizophrenia, we conducted a systematic literature review (SLR) to identify CPGs and synthesize current recommendations for pharmacological management with antipsychotics in the acute and maintenance phases of schizophrenia.

## Results

**Fig. 1:**
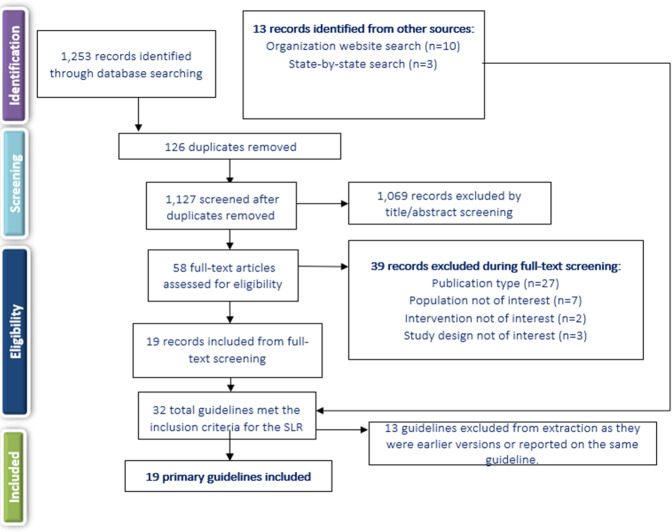
PRISMA diagram. SLR systematic literature review.

Systematic searches for the SLR yielded 1253 hits from the electronic literature databases. After removal of duplicate references, 1127 individual articles were screened at the title and abstract level. Of these, 58 publications were deemed eligible for screening at the full-text level, from which 19 were ultimately included in the SLR. Website searches of relevant organizations yielded 10 additional records, and an additional three records were identified by the state-by-state searches. Altogether, this process resulted in 32 records identified for inclusion in the SLR. Of the 32 sources, 19 primary CPGs, published/issued between 2004 and 2020, were selected for extraction, as illustrated in the PRISMA diagram (Fig. 1). While the most recent APA guideline was identified and available for download in 2020, the reference to cite in the document indicates a publication date of 2021.

Of the 19 included CPGs (Table 1), three had an international focus (from the following organizations: International College of Neuropsychopharmacology [CINP]^22^, United Nations High Commissioner for Refugees [UNHCR]^23^, and World Federation of Societies of Biological Psychiatry [WFSBP]^24–26^); seven originated from the US;^17–19,27–32^ three were from the United Kingdom (British Association for Psychopharmacology [BAP]^33^, the National Institute for Health and Care Excellence [NICE]^34^, and the Scottish Intercollegiate Guidelines Network [SIGN]^35^); and one guideline each was from Singapore^36^, the Polish Psychiatric Association (PPA)^37,38^, the Canadian Psychiatric Association (CPA)^14^, the Royal Australia/New Zealand College of Psychiatrists (RANZCP)^39^, the Association Française de Psychiatrie Biologique et de Neuropsychopharmacologie (AFPBN) from France^40^, and Italy^41^. Fourteen CPGs (74%) recommended treatment with specific antipsychotics and 18 (95%) included recommendations for the use of LAIs, while just seven included a treatment algorithm Table 2). The AGREE II assessment resulted in the highest score across the CPGs domains for NICE^34^ followed by the American Psychiatric Association (APA) guidelines^17^. The CPA^14^, BAP^33^, and SIGN^35^ CPGs also scored well across domains.

**Table 1:** Guidelines included in the review.

*International*	
• International College of Neuropsychopharmacology^22^	• World Federation of Societies of Biological Psychiatry^24–26^
• United Nations High Commissioner for Refugees^23^	
*Country specific*	
United States	Canadian Psychiatric Association^14,77^
• American Association for Community Psychiatrists^29^	France: Association Française de Psychiatrie Biologique et Neuropsychopharmacologie^40^
• American Psychiatric Association^17^	Italy: Italian National Guidelines System^41^
• Florida Medicaid Drug Therapy Management Program^18^	Royal Australia/New Zealand College of Psychiatrists^39^
• New Jersey Division of Mental Health Services^32^	Polish Psychiatric Association ^37,38^
• Oregon Health Authority^19^	Singapore Ministry of Health^36^
• Schizophrenia Patient Outcomes Research^30,31^	
• Texas Medication Algorithm Project^27,28^	
United Kingdom	
• British Association for Psychopharmacology^33^	
• National Institute for Health and Care Excellence^34^	
• Scottish Intercollegiate Guidelines Network^35^

**Table 2:** Characteristics of schizophrenia guidelines.

Guideline	Country	AP class specified (FGA/SGA)	Includes guidelines on dosing	Recommends treatment with specific AP	Includes recommendations for LAIs	Includes a treatment algorithm
APA, 2021^17^	US	Yes	Yes	Yes	Yes	No
Florida Medicaid Program, 2020^18^	US	Yes	Yes	Yes	Yes	Yes
BAP, 2020^33^	UK	No	Yes	No	Yes	No
Oregon Health Authority, 2019^19^	US	Yes	Yes	Yes	Yes	Yes
PPA, 2019^37,38^	Poland	No	Yes	Yes	Yes	Yes
AACP, 2017^29^	US	No	Yes	No	Yes	No
CPA, 2017^14^	Canada	No	Yes	Yes	Yes	No
UNHCR, 2017^23^	International	No	Yes	Yes	Yes	Yes
WFSBP, 2012^24^, 2013^25^, 2017^26^	International	Yes	Yes	Yes	Yes	No
RANZCP, 2016^39^	Australia and New Zealand	Yes	Yes	Yes	Yes	No
NICE, 2014^34^	UK	Yes	Yes	No	Yes	No
AFPBN, 2013^40^	France	Yes	No	No	Yes	No
CINP, 2013^22^	International	No	Yes	Yes	Yes	No
SIGN, 2013^35^	UK	Yes	Yes	Yes	Yes	No
Singapore Ministry of Health, 2011^36^	Singapore	No	Yes	Yes	Yes	Yes
Schizophrenia PORT, 2010^30,31^	US	Yes	Yes	Yes	Yes	No
Italian Guidelines, 2008^41^	Italy	No	No	No	No	No
TMAP, 2008^28^	US	Yes	Yes	Yes	Yes	Yes
NJDMHS, 2005^32^	US	Yes	Yes	Yes	Yes	Yes

*AACP* American Association of Community Psychiatrists, *AFPBN* Association Française de Psychiatrie Biologique et Neuropsychopharmacologie, *AP* antipsychotic, *APA* American Psychiatric Association, *BAP* British Association of Psychopharmacology, *CINP* International College of Neuropsychopharmacology, *CPA* Canadian Psychiatric Association, *LAI* long-acting injectable, *no* not recommended, *NA* not applicable, *NICE* National Institute for Health and Care Excellence, *NJDMHS* New Jersey Division of Mental Health Services, *NR* not reported, *PPA* Polish Psychiatric Association, *PORT* Patient Outcomes Research Team, *RANZCP* Royal Australian/New Zealand College of Psychiatrists, *SIGN* Scottish Intercollegiate Guidelines Network, *TMAP* Texas Medication Algorithm Project, *UNHCR* United Nations High Commissioner for Refugees, *WFSBP* World Federation of Societies of Biological Psychiatry, *yes* recommended.

### Acute therapy

Seventeen CPGs (89.5%) provided treatment recommendations for patients experiencing a first schizophrenia episode^14,17–19,22–24,28,30–36,39–41^, but the depth and focus of the information varied greatly (Supplementary Table 1). In some CPGs, information on treatment of a first schizophrenia episode was limited or grouped with information on treating any acute episode, such as in the CPGs from CINP^22^, AFPBN^40^, New Jersey Division of Mental Health Services (NJDMHS)^32^, the APA^17^, and the PPA^37,38^, while the others provided more detailed information specific to patients experiencing a first schizophrenia episode^14,18,19,23,24,28,33–36,39,41^. The American Association of Community Psychiatrists (AACP) Clinical Tips did not provide any information on the treatment of schizophrenia patients with a first episode^29^.

There was little agreement among CPGs regarding the preferred antipsychotic for a first schizophrenia episode. However, there was strong consensus on antipsychotic monotherapy and that lower doses are generally recommended due to better treatment response and greater adverse effect sensitivity. Some guidelines recommended SGAs over FGAs when treating a first-episode schizophrenia patient (RANZCP^39^, Texas Medication Algorithm Project [TMAP]^28^, Oregon Health Authority^19^), one recommended starting patients on an FGA (UNHCR^23^), and others stated specifically that there was no evidence of any difference in efficacy between FGAs and SGAs (WFSBP^24^, CPA^14^, SIGN^35^, APA^17^, Singapore guidelines^36^), or did not make any recommendation (CINP^22^, Italian guidelines^41^, NICE^34^, NJDMHS^32^, Schizophrenia Patient Outcomes Research Team [PORT]^30,31^). The BAP^33^ and WFBSP^24^ noted that while there was probably no difference between FGAs and SGAs in efficacy, some SGAs (olanzapine, amisulpride, and risperidone) may perform better than some FGAs. The Schizophrenia PORT recommendations noted that while there seemed to be no differences between SGAs and FGAs in short-term studies (≤12 weeks), longer studies (one to two years) suggested that SGAs may provide benefits in terms of longer times to relapse and discontinuation rates^30,31^. The AFPBN guidelines^40^ and Florida Medicaid Program guidelines^18^, which both focus on use of LAI antipsychotics, both recommended an SGA-LAI for patients experiencing a first schizophrenia episode. A caveat in most CPGs was that physicians and their patients should discuss decisions about the choice of antipsychotic and that the choice should consider individual patient factors/preferences, risk of adverse and metabolic effects, and symptom patterns^17–19,22,24,28,30–36,39,41^.

Most CPGs recommended switching to a different monotherapy if the initial antipsychotic was not effective or not well tolerated after an adequate antipsychotic trial at an appropriate dose^14,17–19,22–24,28,32,33,35,36,39^. For patients initially treated with an FGA, the UNHCR recommended switching to an SGA (olanzapine or risperidone)^23^. Guidance on response to treatment varied in the measures used but typically required at least a 20% improvement in symptoms (i.e. reduction in Positive and Negative Syndrome Scale or Brief Psychiatric Rating Scale scores) from pre-treatment levels.

Several CPGs contained recommendations on the duration of antipsychotic therapy after a first schizophrenia episode. The NJDMHS guidelines^32^ recommended nine to 12 months; CINP^22^ recommended at least one year; CPA^14^ recommended at least 18 months; WFSBP^25^, the Italian guidelines^41^, and NICE^34^ recommended 1 to 2 years; and the RANZCP^39^, BAP^33^, and SIGN^35^ recommended at least 2 years. The APA^17^ and TMAP^28^ recommended continuing antipsychotic treatment after resolution of first-episode symptoms but did not recommend a specific length of therapy.

Twelve guidelines^14,18,22,24,28,30,31,33–36,39,40^ (63.2%) discussed the treatment of subsequent/multiple episodes of schizophrenia (i.e., following relapse). These CPGs noted that the considerations guiding the choice of antipsychotic for subsequent/multiple episodes were similar to those for a first episode, factoring in prior patient treatment response, adverse effect patterns and adherence. The CPGs also noted that response to treatment may be lower and require higher doses to achieve a response than for first-episode schizophrenia, that a different antipsychotic than used to treat the first episode may be needed, and that a switch to an LAI is an option.

Several CPGs provided recommendations for patients with specific clinical features (Supplementary Table 1). The most frequently discussed group of clinical features was negative symptoms, with recommendations provided in the CINP^22^, UNHCR^23^, WFSBP^24^, AFPBN^40^, SIGN^35^, BAP^33^, APA^17^, and NJDMHS guidelines;^32^ negative symptoms were the sole focus of the guidelines from the PPA^37,38^. The guidelines noted that due to limited evidence in patients with predominantly negative symptoms, there was no clear benefit for any strategy, but that options included SGAs (especially amisulpride) rather than FGAs (WFSBP^24^, CINP^22^, AFPBN^40^, SIGN^35^, NJDMHS^32^, PPA^37,38^), and addition of an antidepressant (WFSBP^24^, UNHCR^23^, SIGN^35^, NJDMHS^32^) or lamotrigine (SIGN^35^), or switching to another SGA (NJDMHS^32^) or clozapine (NJDMHS^32^). The PPA guidelines^37,38^ stated that the use of clozapine or adding an antidepressant or other medication class was not supported by evidence, but recommended the SGA cariprazine for patients with predominant and persistent negative symptoms, and other SGAs for those with full-spectrum negative symptoms. However, the BAP^33^ stated that no recommendations can be made for any of these strategies because of the quality and paucity of the available evidence.

Some of the CPGs also discussed treatment of other clinical features to a limited degree, including depressive symptoms (CINP^22^, UNHCR^23^, CPA^14^, APA^17^, and NJDMHS^32^), cognitive dysfunction (CINP^22^, UNHCR^23^, WFSBP^24^, AFPBN^40^, SIGN^35^, BAP^33^, and NJDMHS^32^), persistent aggression (CINP^22^, WFSBP^24^, CPA^14^, AFPBN^40^, NICE^34^, SIGN^35^, BAP^33^, and NJDMHS^32^), and comorbid psychiatric diagnoses (CINP^22^, RANZCP^39^, BAP^33^, APA^17^, and NJDMHS^32^).

Fifteen CPGs (78.9%) discussed treatment-resistant schizophrenia (TRS); all defined it as persistent, predominantly positive symptoms after two adequate antipsychotic trials; clozapine was the unanimous first choice^14,17–19,22–24,28,30–36,39^. However, the UNHCR guidelines^23^, which included recommendations for treatment of refugees, noted that clozapine is only a reasonable choice in regions where white blood cell monitoring and specialist supervision are available, otherwise, risperidone or olanzapine are alternatives if they had not been used in the previous treatment regimen.

There were few options for patients who are resistant to clozapine therapy, and evidence supporting these options was limited. The CPA guidelines^14^ therefore stated that no recommendation can be given due to inadequate evidence. Other CPGs discussed options (but noted there was limited supporting evidence), such as switching to olanzapine or risperidone (WFSBP^24^, TMAP^28^), adding a second antipsychotic to clozapine (CINP^22^, NICE^34^, TMAP^28^, BAP^33^, Florida Medicaid Program^18^, Oregon Health Authority^19^, RANZCP^39^), adding lamotrigine or topiramate to clozapine (CINP^22^, Florida Medicaid Program^18^), combination therapy with two non-clozapine antipsychotics (Florida Medicaid Program^18^, NJDMHS^32^), and high-dose non-clozapine antipsychotic therapy (BAP^33^, SIGN^35^). Electroconvulsive therapy was noted as a last resort for patients who did not respond to any pharmacologic therapy, including clozapine, by 10 CPGs^17–19,22,24,28,32,35,36,39^.

### Maintenance therapy

Fifteen CPGs (78.9%) discussed maintenance therapy to various degrees via dedicated sections or statements, while three others referred only to maintenance doses by antipsychotic agent^18,23,29^ without accompanying recommendations (Supplementary Table 2). Only the Italian guideline provided no reference or comments on maintenance treatment. The CINP^22^, WFSBP^25^, RANZCP^39^, and Schizophrenia PORT^30,31^ recommended keeping patients on the same antipsychotic and at the same dose on which they had achieved remission. Several CPGs recommended maintenance therapy at the lowest effective dose (NJDMHS^32^, APA^17^, Singapore guidelines^36^, and TMAP^28^). The CPA^14^ and SIGN^35^ defined the lower dose as 300–400 mg chlorpromazine equivalents or 4–6 mg risperidone equivalents, and the Singapore guidelines^36^ stated that the lower dose should not be less than half the original dose. TMAP^28^ stated that given the relapsing nature of schizophrenia, the maintenance dose should often be close to the original dose. While SIGN^35^ recommended that patients remain on the same antipsychotic that provided remission, these guidelines also stated that maintenance with amisulpride, olanzapine, or risperidone was preferred, and that chlorpromazine and other low-potency FGAs were also suitable. The BAP^33^ recommended that the current regimen be optimized before any dose reduction or switch to another antipsychotic occurs. Several CPGs recommended LAIs as an option for maintenance therapy (see next section).

Altogether, 10/18 (55.5%) CPGs made no recommendations on the appropriate duration of maintenance therapy, noting instead that each patient should be considered individually. Other CPGs made specific recommendations: Both the Both BAP^33^ and SIGN^35^ guidelines suggested a minimum of 2 years, the NJDMHS guidelines^32^ recommended 2–3 years; the WFSBP^25^ recommended 2–5 years for patients who have had one relapse and more than 5 years for those who have had multiple relapses; the RANZCP^39^ and the CPA^14^ recommended 2–5 years; and the CINP^22^ recommended that maintenance therapy last at least 6 years for patients who have had multiple episodes. The TMAP was the only CPG to recommend that maintenance therapy be continued indefinitely^28^.

### Recommendations on the use of LAIs

All CPGs except the one from Italy (94.7%) discussed the use of LAIs for patients with schizophrenia to some extent. As shown in Table 3, among the 18 CPGs, LAIs were primarily recommended in 14 CPGs (77.8%) for patients who are non-adherent to other antipsychotic administration routes (CINP^22^, UNHCR^23^, RANZCP^39^, PPA^37,38^, Singapore guidelines^36^, NICE^34^, SIGN^35^, BAP^33^, APA^17^, TMAP^28^, NJDMHS^32^, AACP^29^, Oregon Health Authority^19^, Florida Medicaid Program^18^). Twelve CPGs (66.7%) also noted that LAIs should be prescribed based on patient preference (RANZCP^39^, CPA^14^, AFPBN^40^, Singapore guidelines^36^, NICE^34^, SIGN^35^, BAP^33^, APA^17^, Schizophrenia PORT^30,31^, AACP^29^, Oregon Health Authority^19^, Florida Medicaid Program^18^).

**Table 3:** Recommendations for LAI antipsychotics in the treatment of schizophrenia.

Guideline	Use for a first- episode schizophrenia	Use for maintenance treatment	Use for nonadherence	Use based on patient preference
APA, 2021^17^	Not reported	Not reported	Yes	Yes
Florida Medicaid Program, 2020^18^	Yes	Yes	Yes	Yes
BAP, 2020^33^	No	Yes	Yes	Yes
Oregon Health Authority, 2019^19^	Not reported	Yes	Yes	Yes
PPA, 2019^37,38^	Not reported	Not reported	Yes	Not reported
AACP, 2017^29^	Not reported	Yes	Yes	Yes
CPA, 2017^14^	Not reported	Not reported	Not reported	Yes
UNHCR, 2017^23^	Not reported	Not reported	Yes	Not reported
WFSBP, 2012^24^, 2013^25^, 2017^26^	Not reported	Yes	Not reported	Not reported
RANZCP, 2016^39^	Yes	Yes	Yes	Yes
NICE, 2014^34^	No	Yes	Yes	Yes
AFPBN, 2013^40^	Yes	Yes	Not reported	Yes
CINP, 2013^22^	Not reported	Not reported	Yes	Not reported
SIGN, 2013^35^	No	Yes	Yes	Yes
Singapore Ministry of Health, 2011^36^	No	Yes	Yes	Yes
Schizophrenia PORT, 2010^30,31^	No	Yes	Not reported	Yes
Italian Guidelines, 2008^41^	NA	NA	NA	NA
TMAP, 2008^28^	Yes	Yes	Yes	Not reported
NJDMHS, 2005^32^	Yes	Yes	Yes	Not reported

*AACP* American Association of Community Psychiatrists, *AFPBN* Association Française de Psychiatrie Biologique et Neuropsychopharmacologie, *APA* American Psychiatric Association, *BAP* British Association of Psychopharmacology, *CINP* International College of Neuropsychopharmacology, *CPA* Canadian Psychiatric Association, *LAI* long-acting injectable, *no* not recommended, *NA* not applicable, *NICE* National Institute for Health and Care Excellence, *NJDMHS* New Jersey Division of Mental Health Services, *PPA* Polish Psychiatric Association, *PORT* Patient Outcomes Research Team, *RANZCP* Royal Australian/New Zealand College of Psychiatrists, *SIGN* Scottish Intercollegiate Guidelines Network, *TMAP* Texas Medication Algorithm Project, *UNHCR* United Nations High Commissioner for Refugees, *WFSBP* World Federation of Societies of Biological Psychiatry, *yes* recommended.

Thirteen CPGs (72.2%) recommended LAIs as maintenance therapy^18,19,24,28–36,39,40^. While five CPGs (27.8%), i.e., AFPBN^40^, RANZCP^39^, TMAP^28^, NJDMHS^32^, and the Florida Medicaid Program^18^ recommended LAIs specifically for patients experiencing a first episode. While the CPA^14^ did not make any recommendations regarding when LAIs should be used, they discussed recent evidence supporting their use earlier in treatment. Five guidelines (27.8%, i.e., Singapore^36^, NICE^34^, SIGN^35^, BAP^33^, and Schizophrenia PORT^30,31^) noted that evidence around LAIs was not sufficient to support recommending their use for first-episode patients. The AFPBN guidelines^40^ also stated that LAIs (SGAs as first-line and FGAs as second-line treatment) should be more frequently considered for maintenance treatment of schizophrenia. Four CPGs (22.2%, i.e., CINP^22^, UNHCR^23^, Italian guidelines^41^, PPA guidelines^37,38^) did not specify when LAIs should be used. The AACP guidelines^29^, which evaluated only LAIs, recommended expanding their use beyond treatment for nonadherence, suggesting that LAIs may offer a more convenient mode of administration or potentially address other clinical and social challenges, as well as provide more consistent plasma levels.

### Treatment algorithms

Only Seven CPGs (36.8%) included an algorithm as part of the treatment recommendations. These included decision trees or flow diagrams that map out initial therapy, durations for assessing response, and treatment options in cases of non-response. However, none of these guidelines defined how to measure response, a theme that also extended to guidelines that did not include treatment algorithms. Four of the seven guidelines with algorithms recommended specific antipsychotic agents, while the remaining three referred only to the antipsychotic class.

LAIs were not consistently incorporated in treatment algorithms and in six CPGs were treated as a separate category of medicine reserved for patients with adherence issues or a preference for the route of administration. The only exception was the Florida Medicaid Program^18^, which recommended offering LAIs after oral antipsychotic stabilization even to patients who are at that point adherent to oral antipsychotics.

### Benefits and harms

The need to balance the efficacy and safety of antipsychotics was mentioned by all CPGs as a basic treatment paradigm.

Ten CPGs provided conclusions on benefits of antipsychotic therapy. The APA^17^ and the BAP^33^ guidelines stated that antipsychotic treatment can improve the positive and negative symptoms of psychosis and leads to remission of symptoms. These CPGs^17,33^ as well as those from NICE^34^ and CPA^14^ stated that these treatment effects can also lead to improvements in quality of life (including quality-adjusted life years), improved functioning, and reduction in disability. The CPA^14^ and APA^17^ guidelines noted decreases in hospitalizations with antipsychotic therapy, and the APA guidelines^17^ stated that long-term antipsychotic treatment can also reduce mortality. The UNHCR^23^ and the Italian^41^ guidelines noted that early intervention increased positive outcomes. The WFSBP^24^, AFPBN^40^, CPA^14^, BAP^33^, APA^17^, and NJDMHS^32^ affirmed that relapse prevention is a benefit of continued/maintenance treatment.

Some CPGs (WFSBP^24^, Italian^41^, CPA^14^, and SIGN^35^) noted that reduced risk for extrapyramidal adverse effects and treatment discontinuation were potential benefits of SGAs vs. FGAs.

The risk of adverse effects (e.g., extrapyramidal, metabolic, cardiovascular, and hormonal adverse effects, sedation, and neuroleptic malignant syndrome) was noted by all CPGs as the major potential harm of antipsychotic therapy^14,17–19,22–24,28–32,34–37,39–42^. These adverse effects are known to limit long-term treatment and adherence^24^.

## Discussion

This SLR of CPGs for the treatment of schizophrenia yielded 19 most updated versions of individual CPGs, published/issued between 2004 and 2020. Structuring our comparative review according to illness phase, antipsychotic type and formulation, response to antipsychotic treatment as well as benefits and harms, several areas of consistent recommendations emerged from this review (e.g., balancing risk and benefits of antipsychotics, preferring antipsychotic monotherapy; using clozapine for treatment-resistant schizophrenia). On the other hand, other recommendations regarding other areas of antipsychotic treatment were mostly consistent (e.g., maintenance antipsychotic treatment for some time), somewhat inconsistent (e.g., differences in the management of first- vs multi-episode patients, type of antipsychotic, dose of antipsychotic maintenance treatment), or even contradictory (e.g., role of LAIs in first-episode schizophrenia patients).

Consistent with RCT evidence^43,44^, antipsychotic monotherapy was the treatment of choice for patients with first-episode schizophrenia in all CPGs, and all guidelines stated that a different single antipsychotic should be tried if the first is ineffective or intolerable. Recommendations were similar for multi-episode patients, but factored in prior patient treatment response, adverse effect patterns, and adherence. There was also broad consensus that the side-effect profile of antipsychotics is the most important consideration when making a decision on pharmacologic treatment, also reflecting meta-analytic evidence^4,5,10^. The risk of extrapyramidal symptoms (especially with FGAs) and metabolic effects (especially with SGAs) were noted as key considerations, which are also reflected in the literature as relevant concerns^4,45,46^, including for quality of life and treatment nonadherence^47–50^.

Largely consistent with the comparative meta-analytic evidence regarding the acute^4,51,52^ and maintenance antipsychotic treatment^5^ effects of schizophrenia, the majority of CPGs stated there was no difference in efficacy between SGAs and FGAs (WFSBP^24^, CPA^14^, SIGN^35^, APA^17^, and Singapore guidelines^36^), or did not make any recommendations (CINP^22^, Italian guidelines^41^, NICE^34^, NJDMHS^32^, and Schizophrenia PORT^30,31^); three CPGs (BAP^33^, WFBSP^24^, and Schizophrenia PORT^30,31^) noted that SGAs may perform better than FGAs over the long term, consistent with a meta-analysis on this topic^53^.

The 12 CPGs that discussed treatment of subsequent/multiple episodes generally agreed on the factors guiding the choices of an antipsychotic, including that the decision may be more complicated and response may be lower than with a first episode, as described before^7,54–56^.

There was little consensus regarding maintenance therapy. Some CPGs recommended the same antipsychotic and dose that achieved remission (CINP^22^, WFSBP^25^, RANZCP^39^, and Schizophrenia PORT^30,31^) and others recommended the lowest effective dose (NJDMHS^32^, APA^17^, Singapore guidelines^36^, TMAP^28^, CPA^14^, and SIGN^35^). This inconsistency is likely based on insufficient data as well as conflicting results in existing meta-analyses on this topic^57–59^.

The 15 CPGs that discussed TRS all used the same definition for this condition, consistent with recent commendations^60^, and agreed that clozapine is the primary evidence-based treatment choice^14,17–19,22–24,28,30–36,39^, reflecting the evidence base^61–63^. These CPGs also agreed that there are few options well supported by evidence for patients who do not respond to clozapine, with a recent meta-analysis of RCTs showing that electroconvulsive therapy augmentation may be the most evidence-based treatment option^64^.

One key gap in the treatment recommendations was how long patients should remain on antipsychotic therapy after a first episode or during maintenance therapy. While nine of the 17 CPGs discussing treatment of a first episode provided a recommended timeframe (varying from 1 to 2 years)^14,22,24,32–35,39,41^, the APA^17^ and TMAP^28^ recommended continuing antipsychotic treatment after resolution of first-episode symptoms but did not recommend a specific length of therapy. Similarly, six of the 18 CPGs discussing maintenance treatment recommended a specific duration of therapy (ranging from two to six years)^14,22,25,32,39^, while as many as 10 CPGs did not point to a firm end of the maintenance treatment, instead recommending individualized decisions. The CPGs not stating a definite endpoint or period of maintenance treatment after repeated schizophrenia episodes or even after a first episode of schizophrenia, reflects the different evidence types on which the recommendation is based. The RCT evidence ends after several years of maintenance treatment vs. discontinuation supporting ongoing antipsychotic treatment; however, naturalistic database studies do not indicate any time period after which one can safely discontinue maintenance antipsychotic care, even after a first schizophrenia episode^8,65^. In fact, stopping antipsychotics is associated not only with a substantially increased risk of hospitalization but also mortality^65–67^. In this sense, not stating an endpoint for antipsychotic maintenance therapy should not be taken as an implicit statement that antipsychotics should be discontinued at any time; data suggest the contrary.

A further gap exists regarding the most appropriate treatment of negative symptoms, such as anhedonia, amotivation, asociality, affective flattening, and alogia^1^, a long-standing challenge in the management of patients with schizophrenia. Negative symptoms often persist in patients after positive symptoms have resolved, or are the presenting feature in a substantial minority of patients^22,35^. Negative symptoms can also be secondary to pharmacotherapy^22,68^. Antipsychotics have been most successful in treating positive symptoms, and while eight of the CPGs provided some information on treatment of negative symptoms, the recommendations were generally limited^17,22–24,32,33,35,40^. Negative symptom management was a focus of the PPA guidelines, but the guidelines acknowledged that supporting evidence was limited, often due to the low number of patients with predominantly negative symptoms in clinical trials^37,38^. The Polish guidelines are also one of the more recently developed and included the newer antipsychotic cariprazine as a first-line option, which although being a point of differentiation from the other guidelines, this recommendation was based on RCT data^69^.

Another area in which more direction is needed is on the use of LAIs. While all but one of the 19 CPGs discussed this topic, the extent of information and recommendations for LAI use varied considerably. All CPGs categorized LAIs as an option to improve adherence to therapy or based on patient preference. However, 5/18 CPGs (27.8%) recommended the use of LAI early in treatment (at first episode: AFPBN^40^, RANZCP^39^, TMAP^28^, NJDMHS^32^, and Florida Medicaid Program^18^) or across the entire illness course, while five others stated there was not sufficient evidence to recommend LAIs for these patients (Singapore^36^, NICE^34^, SIGN^35^, BAP^33^, and Schizophrenia PORT^30,31^). The role of LAIs in first-episode schizophrenia was the only point where opposing recommendations were found across CPGs. This contradictory stance was not due to the incorporation of newer data suggesting benefits of LAIs in first episode and early-phase patients with schizophrenia^70–74^ in the CPGs recommending LAI use in first-episode patients, as CPGs recommending LAI use were published between 2005 and 2020, while those opposing LAI use were published between 2011 and 2020. Only the Florida Medicaid CPG recommended LAIs as a first step equivalent to oral antipsychotics (OAP) after initial OAP response and tolerability, independent of nonadherence or other clinical variables. This guideline was also the only CPG to fully integrate LAI use in their clinical algorithm. The remaining six CPGs that included decision tress or treatment algorithms regarded LAIs as a separate paradigm of treatment reserved for nonadherence or patients preference rather than a routine treatment option to consider. While some CPGs provided fairly detailed information on the use of LAIs (AFPBN^40^, AACP^29^, Oregon Health Authority^19^, and Florida Medicaid Program^18^), others mentioned them only in the context of adherence issues or patient preference. Notably, definitions of and means to determine nonadherence were not reported. One reason for this wide range of recommendations regarding the placement of LAIs in the treatment algorithm and clinical situations that prompt LAI use might be due to the fact that CPGs generally favor RCT evidence over evidence from other study designs. In the case of LAIs, there was a notable dissociation between consistent meta-analytic evidence of statistically significant superiority of LAIs vs OAPs in mirror-image^75^ and cohort study designs^76^ and non-significant advantages in RCTs^77^. Although patients in RCTs comparing LAIs vs OAPs were less severely ill and more adherent to OAPs^77^ than in clinical care and although mirror-image and cohort studies arguably have greater external validity than RCTs^78^, CPGs generally disregard evidence from other study designs when RCT evidence exits. This narrow focus can lead to disregarding important additional data. Nevertheless, a most updated meta-analysis of all 3 study designs comparing LAIs with OAPs demonstrated consistent superiority of LAIs vs OAPs for hospitalization or relapse across all 3 designs^79^, which should lead to more uniform recommendations across CPGs in the future.

Only seven CPGs included treatment algorithms or flow charts to guide LAI treatment selection for patients with schizophrenia^17–19,24,29,35,40^. However, there was little commonality across algorithms beyond the guidance on LAIs mentioned above, as some listed specific treatments and conditions for antipsychotic switches, while others indicated that medication choice should be based on a patient’s preferences and responses, side effects, and in some cases, cost effectiveness. Since algorithms and flow charts facilitate the reception, adoption and implementation of guidelines, future CPGs should include them as dissemination tools, but they need to reflect the data and detailed text and be sufficiently specific to be actionable.

The systematic nature in the identification, summarization, and assessment of the CPGs is a strength of this review. This process removed any potential bias associated with subjective selection of evidence, which is not reproducible. However, only CPGs published in English were included and regardless of their quality and differing timeframes of development and publication, complicating a direct comparison of consensus and disagreement. Finally, based on the focus of this SLR, we only reviewed pharmacologic management with antipsychotics. Clearly, the assessment, other pharmacologic and, especially, psychosocial interventions are important in the management of individuals with schizophrenia, but these topics that were covered to varying degrees by the evaluated CPGs were outside of the scope of this review.

Numerous guidelines have recently updated their recommendations on the pharmacological treatment of patients with schizophrenia, which we have summarized in this review. Consistent recommendations were observed across CPGs in the areas of balancing risk and benefits of antipsychotics when selecting treatment, a preference for antipsychotic monotherapy, especially for patients with a first episode of schizophrenia, and the use of clozapine for treatment-resistant schizophrenia. By contrast, there were inconsistencies with regards to recommendations on maintenance antipsychotic treatment, with differences existing on type and dose of antipsychotic, as well as the duration of therapy. However, LAIs were consistently recommended, but mainly suggested in cases of nonadherence or patient preference, despite their established efficacy in broader patient populations and clinical scenarios in clinical trials. Guidelines were sometimes contradictory, with some recommending LAI use earlier in the disease course (e.g., first episode) and others suggesting they only be reserved for later in the disease. This inconsistency was not due to lack of evidence on the efficacy of LAIs in first-episode schizophrenia or the timing of the CPG, so that other reasons might be responsible, including possibly bias and stigma associated with this route of treatment administration. Lastly, gaps existed in the guidelines for recommendations on the duration of maintenance treatment, treatment of negative symptoms, and the development/use of treatment algorithms whenever evidence is sufficient to provide a simplified summary of the data and indicate their relevance for clinical decision making, all of which should be considered in future guideline development/revisions.

## Methods

The SLR followed established best methods used in systematic review research to identify and assess the available CPGs for pharmacologic treatment of schizophrenia with antipsychotics in the acute and maintenance phases^80,81^. The SLR was conducted in accordance with the Preferred Reporting Items for Systematic Reviews and Meta-Analyses (PRISMA) guidelines, including use of a prespecified protocol to outline methods for conducting the review. The protocol for this review was approved by all authors prior to implementation but was not submitted to an external registry.

### Data sources and search algorithms

Searches were conducted by two independent investigators in the MEDLINE and Embase databases via OvidSP to identify CPGs published in English. Articles were identified using search algorithms that paired terms for schizophrenia with keywords for CPGs. Articles indexed as case reports, reviews, letters, or news were excluded from the searches. The database search was limited to CPGs published from January 1, 2004, through December 19, 2019, without limit to geographic location. In addition to the database sources, guideline body websites and state-level health departments from the US were also searched for relevant CPGs published through June 2020. A manual check of the references of recent (i.e., published in the past three years), relevant SLRs and relevant practice CPGs was conducted to supplement the above searches and ensure and the most complete CPG retrieval.

### Ethics

This study did not involve human subjects as only published evidence was included in the review; ethical approval from an institution was therefore not required.

### Selection of CPGs for inclusion

Each title and abstract identified from the database searches was screened and selected for inclusion or exclusion in the SLR by two independent investigators based on the populations, interventions/comparators, outcomes, study design, time period, language, and geographic criteria shown in Table 4. During both rounds of the screening process, discrepancies between the two independent reviewers were resolved through discussion, and a third investigator resolved any disagreement. Articles/documents identified by the manual search of organizational websites were screened using the same criteria. All accepted studies were required to meet all inclusion criteria and none of the exclusion criteria. Only the most recent version of organizational CPGs was included for data extraction.

**Table 4:** Study selection criteria.

Domain	Inclusion	Exclusion
Population	Adult diagnosed with schizophrenia	Disorders other than schizophrenia
Intervention/comparator	Any pharmacotherapy	Lack of guidance on pharmacotherapy
Outcomes	Guidelines related to the treatment of the acute and maintenance phases of schizophrenia	None of the outcomes listed in the inclusion criteria are reported
Study design	Clinical practice treatment guidelines	Meta-analyses, SLRs, RCTs, single-arm trials, non-randomized trials, retrospective and prospective observational studies, case reports, reviews, news, commentary and letters
Time period	Literature Databases: January 1, 2004, through December 19, 2019	CPGs published in journals before 2004 or after December 2019
	Guideline body websites and state-level health departments from the US: June 2020	CPGs available on guideline body websites after June 2020^a^
Language	English	Languages other than English
Geographic Location	Overarching geographic guidelines	Region-specific guidelines

*CPG* clinical practice guideline, *RCT* randomized controlled trial, *SLR* systematic literature review.

^a^The draft of the third version of the APA guideline became available on their website in December of 2019, but the final version was not published until late 2020 and the final copyright reflects a 2021 date.

### Data extraction and synthesis

Information on the recommendations regarding the antipsychotic management in the acute and maintenance phases of schizophrenia and related benefits and harms was captured from the included CPGs. Each guideline was reviewed and extracted by a single researcher and the data were validated by a senior team member to ensure accuracy and completeness. Additionally, each included CPG was assessed using the Appraisal of Guidelines for Research and Evaluation II (AGREE II) tool. Following extraction and validation, results were qualitatively summarized across CPGs.

### Reporting summary

Further information on research design is available in the Nature Research Reporting Summary linked to this article.

## Supplementary information

Supplementary Information

Reporting Summary

## Data Availability

The data that support the findings of the SLR are available from the corresponding author upon request.
