# Supplementary material for: Systematic literature review of schizophrenia clinical practice guidelines on acute and maintenance management with antipsychotics
Source: Schizophrenia (Heidelb). 2022 Feb 24;8(1):5. doi: 10.1038/s41537-021-00192-x (PMC8873492; doi:10.1038/s41537-021-00192-x)
Supplement: Supplementary file 1 — Supplementary Information [file 41537_2021_192_MOESM1_ESM.docx]

**Supplemental Table 1. Recommendations by episode, antipsychotic agent, and for patients with negative symptoms or depression**

| Guideline, Country | First episode vs multi-episode | Choice of AP (type and mono-vs polypharmacy) | Negative symptoms | Depression |
| --- | --- | --- | --- | --- |
| APA, 2021,^17^ US | There were no specific recommendations for treatment of a first schizophrenia episode.  Instead, the guideline focused on acute treatment. The initial dose of medication will depend on such factors as the medication formulation, the characteristics of the patient, and whether a prior trial of AP medication has occurred. With the exception of clozapine, the dose of most AP medications can be increased relatively quickly to a typical therapeutic dose once an initial dose has been tolerated. | The choice of an AP agent depends on many factors that are specific to an individual patient. Thus, before initiating treatment with AP medication, it is recommended that as part of selecting a medication, the treating clinician gather information on the patient’s treatment-related preferences and prior treatment responses and then discuss with the patient the potential benefits and risks of medication as compared with other management options.  An evidence-based ranking of FGAs and SGAs or an algorithmic approach to AP selection is not possible because of the significant heterogeneity in clinical trial designs, the limited numbers of head-to-head comparisons of AP medications, and the limited clinical trial data for a number of the AP medications. By the same token, it is not possible to note a preference for either SGAs or FGAs | There were no specific guideline statements regarding negative symptoms. Supporting evidence showed that treatment with APs led to improvements in negative symptoms. | Depressive symptoms are common in individuals with schizophrenia and should be addressed as part of treatment planning. The approach to treating depression will be grounded in a careful differential diagnosis that considers the possible contributions of demoralization, negative symptoms of schizophrenia, side effects of AP medications, substance intoxication or withdrawal, physical health condition, or a co-occurring major depressive episode. Depressive symptoms that occur during an acute episode of psychosis often improve as psychotic symptoms respond to treatment. |
| Florida Medicaid Program, 2020,^18^ US | Initial treatment should include monotherapy with an AP (SGA) other than clozapine—either oral, or oral AP followed by the same SGA-LAI (if tolerable and sufficiently efficacious). Consider lower doses for first episode due to better response and higher side effects to medications in pharmaceutically-naïve patients.  If initial trial of AP monotherapy is unsuccessful, try monotherapy with another SGA (either oral or LAI) with low metabolic adverse effects. | Following initial treatment, after stabilization or obtaining sufficient evidence of efficacy and tolerability any of the following LAIs can be offered:  - Aripiprazole monohydrate  - Aripiprazole lauroxil  - Paliperidone palmitate  - Risperidone microspheres  - Risperidone extended release subcutaneous injectable.  Practitioners should try to avoid polypharmacy with different APs, as the evidence for efficacy and safety is lacking; however, this can be an option when treatment resistance exists and clozapine has not been effective. | NR | NR |
| BAP, 2020,^33^ UK | First-episode: AP medication is the first line of treatment for psychosis. Initial doses should be below those typical in established illness but sufficient for efficacy. For later acute episodes, choice of AP drug should be based on the same criteria as suggested for first episode, but should additionally take into account:  -Any preference a patient may have for any particular AP medication.  -A patient’s past experience of individual AP medications in terms of relief of symptoms and side effects, including aversive subjective experiences. | The data remain insufficient to allow for any clear recommendation of superiority for any individual AP medication. | If a trial of medication for negative symptoms is to be attempted, it would be advisable to use one of the newer rating scales for negative symptoms (CAINS or BNSS) to monitor treatment response, with a plan to discontinue treatment if no clinically meaningful response is seen. | Clinicians would be justified in discussing an individual therapeutic trial of antidepressant medication with their patients with schizophrenia who are depressed. Antidepressant medication should be discontinued if no benefit is found after an adequate trial. |
| Oregon Health Authority, 2019,^19^ UK | SGAs aripiprazole or risperidone are recommended as initial treatment. The treatment choice is based on avoidance of side potential side effects. Risperidone is prescribed to minimize the risk of akathisia and treatment emergent activation or agitation. Aripiprazole is prescribed to minimize risk of weight gain and diabetes, pseudoparkinsonism and tardive dyskinesia. Adequate response should be achieved in 2-4 weeks. If FGA is the preferred medication, due to history of tolerability, predominant positive symptoms or to minimize cardiometabolic risks, fluphenazine or haloperidol were recommended. | Monotherapy was recommended either aripiprazole or risperidone among the SGAs and fluphenazine or haloperidol from the FGAs. | NR | A patient with psychosis and low energy or depression may benefit more from a medication with more activating effects. |
| PPA, 2019,^37,38^ Poland | Treatment of patients with predominantly negative symptoms should involve tailoring the therapy to the phase (acute episode, consolidation, stabilization, and remission) via:  i. monitoring psychopathological symptoms and adverse events;  ii. implementing the therapy plan with a given medication according to the treatment algorithm. | Recommendations focus on treatment of patients with predominantly negative symptoms.  Amisulpride, olanzapine, paliperidone, and risperidone were all found to be efficacious in trials of patients with predominantly negative symptoms.  Cariprazine was the only medication among those explored that demonstrated a statistically significant advantage over another second-generation medication in a trial population of patients with predominant negative symptoms.  Treatment with quetiapine, lurasidone, clozapine, and quetiapine, sertindole, and ziprasidone were also included in the treatment algorithm, but evidence on the efficacy of those treatments was demonstrated in trials from the general schizophrenia population. | In pharmacotherapy standards two populations of schizophrenia patients with negative symptoms should be distinguished: individuals with negative symptoms, as well as patients with predominant and persistent negative symptoms. In order to ensure optimal conditions of pharmacological therapy, it is necessary to establish an individual therapeutic plan. The treatment should be comprised of many stages and modified depending on both the achieved and unachieved goals. | Adding antidepressants to AP therapy has been a common practice for a long time. Any possible mood enhancement is to reduce at least secondary negative symptoms. But there are few convincing, methodologically advanced papers that would confirm such concepts experimentally. What can encourage the use of AP and antidepressant combinations is the observed significant reduction of mortality among patients treated with them. |
| AACP, 2017,^29^ US | AACP recommends expanding the scope of indications for LAIs well beyond the single indication of AP nonadherence. LAIs may be extremely helpful to many individuals in facilitating a more convenient way to take medications and/or better addressing a variety of clinical and social challenges.  The following factors may lead psychiatrists to consider offering LAIs:  -Personal preference of the person receiving care  -Past history of positive response to long-acting AP  -Past history of non-adherence to oral medications  -Homelessness  -Criminal Justice involvement (to demonstrate or promote adherence)  -Frequent utilization of ED and/or hospital  -Co-occurring substance use disorder  -Cognitive challenges  -Anosognosia or limited insight  -Facilitating a smooth transition to clozapine | NR | NR | NR |
| CPA, 2017,^39^ Canada | Choice of AP medication should be made by the patient and physician together, taking into account views of a carer where appropriate. Provide information and discuss the likely benefits and side effects of each drug. The inconsistency of findings argues against established clinical superiority for a specific AP in first-episode schizophrenia. Following an acute episode of schizophrenia, individuals should be offered maintenance treatment with AP medication at low or moderate regular dosing. | AP choice for first-episode is recommended to be made by the patient and physician together. For maintenance therapy, AP medication at low or moderate regular dosing of around 300 to 400 mg of chlorpromazine equivalents, 4 to 6 mg of risperidone, or other equivalents daily is recommended. | NR | Comorbid Depressive Symptoms Individuals who meet criteria for depressive disorder should be treated according to relevant clinical practice guidelines for depression, including the use of antidepressant. |
| UNHCR, 2017,^23^ International | In humanitarian non-specialized settings treatments were recommended by line of therapy with FGAs recommended as first-line treatment followed by SGAs. | Firstline treatment: Haloperidol or chlorpromazine  Second-line treatment: Risperidone or olanzapine  Third-line treatment: switch to risperidone or olanzapine as an alternative approach to second-line or consider clozapine if specialist monitoring is available. | NR | NR |
| WFSBP, 2012^24^, 2013^25^, 2017,^26^ International | FGAs and SGAs are both effective in the treatment of first-episode schizophrenia. Patients suffering from their first episode should be treated with lower AP dosages than chronically ill patients. Due to the reduced risk of inducing neurological side effects, the first-line use of SGAs in first episode schizophrenia patients are recommended with limited evidence.  FGAs and SGAs are both effective in the treatment of acute relapse. All established FGAs and SGAs can be used in the treatment of acute schizophrenia. Each AP selection procedure must be undertaken individually, respecting the patient’s experience with certain drug classes and the individual side effect profile | Olanzapine, risperidone and quetiapine are the best approved SGAs in first-episode patient. Haloperidol is the best approved FGA in first episode patients. | For the treatment of secondary negative symptoms, both FGAs and SGAs have a modest efficacy. For primary negative symptoms treatment with certain SGAs (amisulpride, aripiprazole, clozapine, olanzapine, quetiapine, ziprasidone), but not with FGAs, is recommended with inconsistent evidence and with the need for more studies to prove the efficacy.  There is some limited evidence for the efficacy of antidepressants in the treatment of negative symptoms. | Depressive symptoms should be quantified by using the Calgary Depression Scale for Schizophrenia and it is not recommended to switch APs or to introduce an antidepressant immediately. It has been established that depressive symptoms may improve in the course of time as psychotic symptoms improve and this effect of AP treatment should be waited for first. In case of persistent depressive symptoms, certain SGAs (e.g. amisulpride, aripiprazole, clozapine, olanzapine and quetiapine) seem to be superior to others (e.g., risperidone). The introduction of antidepressants may be warranted in cases where the criteria for a major depressive episode are fulfilled. When adding antidepressants a close monitoring of psychotic symptoms, suicidality, drug-drug interactions and ECG is necessary. Lithium is also recommended, whereas the efficacy has not been shown in all studies. |
| RANZCP, 2016,^40^Australia and New Zealand | For a first-episode the lowest effective AP dose should be used to establish treatment acceptance and minimize side effects. The management plan should be discussed fully with the individual and their family/carers, wherever possible. The benefits and risks of drug therapy should be explained in a non-coercive manner. LAI AP agents should be offered to patients early in the clinical course of schizophrenia.  Management of an acute relapse should involve adequate sequential trials (at least 6 weeks of 300–1000 mg in chlorpromazine equivalents) of two AP medications, of which at least one should be a SGA, should have been conducted. If there has been poor or uncertain adherence, or it is the individual’s preference, LAI AP medication should be considered. | Oral SGAs should be prescribed as first- and second-line treatment for people with a first episode. The initial dose should be low. If response is slow or incomplete, the dose should be increased slowly at suitable intervals.  The choice of AP medicines should be based on:  -the individual’s preference after risks and potential benefits have been explained,  -the person’s prior response to the medicine (if known),  -clinical response to an adequate treatment trial,  -individual tolerability,  -potential long-term adverse effects  Prescribe only one AP agent at a time, unless it has been clearly demonstrated that the person’s symptoms are resistant to monotherapy. | NR | NR |
| NICE, 2014,^34^ UK | For people with first episode psychosis offer: oral AP medication in conjunction with psychological interventions.  For people with an acute exacerbation or recurrence of psychosis or schizophrenia, offer: oral AP medication in conjunction with psychological interventions. | The choice of AP medication should be made by the service user and healthcare professional together, taking into account the views of the carer if the service user agrees. Provide information and discuss the likely benefits and possible side effects of each drug, including: metabolic, extrapyramidal, cardiovascular, hormonal, and other.  For people with an acute exacerbation or recurrence of psychosis or schizophrenia, offer oral AP medication or review existing medication. The choice of drug should be influenced by the same criteria recommended for starting treatment. Take into account the clinical response and side effects of the service user’s current and previous medication. | NR | NR |
| AFPBN, 2013,^41^ France | Guideline focused on recommendations for LAIs.  Only LAI SGA are considered as a therapeutic option during the initial phase of schizophrenic illness:  – They are recommended from the first psychotic episode.  – Their introduction from the first recurrent psychotic episode is also recommended (if the patient was not treated with an LAI AP). | LAI FGA are not recommended during the early course of schizophrenia (i.e. in a patient who has been newly diagnosed with schizophrenia and who has had no previous AP treatment).  LAI SGA are recommended (in monotherapy or combination) as 1st line treatment.  - LAI FGA are recommended (in monotherapy or combination) as 2nd line treatment.  The preferential choice criteria for an LAI formulation (as first line treatment) in patients with schizophrenia are:  -poor adherence/non-acceptance  -patient preference  -patients presenting with dangerous behavior  -socially/family isolated patients  -Patients with cognitive impairment with an impact on their functioning | The prevalence of positive or negative symptoms is not a specific factor in choosing to use a depot treatment.  If a depot treatment is chosen, only LAI SGA are recommended (as 2nd line treatment) in cases of predominant negative symptoms. | NR |
| CINP, 2013,^22^ International | First episode patients overall respond better to AP drugs than chronic patients and seem to need lower doses, which could be targeted at the lower end of officially registered ranges. Side-effects may therefore play an even higher role in choosing among APs than in more chronic patients, although some evidence suggests in part similar efficacy differences between drugs as in chronic populations. | For acute episodes, patients should be started on monotherapy with one drug. No strong evidence supports the use of polypharmacy over monotherapy for efficacy.  Choice of AP should be guided by consideration of the efficacy and side effect profile of treatments as well as patient preference, evidence of prior response to the same drug, and to avoid side effects experienced with a drug in the past. | APs reduce general negative symptoms, but the evidence for negative symptoms that persist after acute treatment is limited. Rule out secondary negative symptoms due to extrapyramidal side-effects (e.g., by lowering the dose, a trial of anticholinergic medication, or switching to an AP with a low EPS risk). Avoid secondary negative symptoms by using a drug with a low EPS risk. Low-dose amisulpride (50-300mg/day) is the best studied drug for this indication and is the only one that has been shown to be more efficacious than placebo in several trials. Adding an antidepressant can be effective. | Depressive symptoms associated with an acute episode of schizophrenia should not be automatically treated with an antidepressant, because they may resolve with the treatment with an AP alone. If there is a suspicion of AP induced depressive symptoms, these should be ruled out by dose reduction, anticholinergic medication or by switching to a drug with a lower EPS risk. Persistent or post-psychotic depression may be treated with an antidepressant added to the AP. The risk to provoke an exacerbation by adding an antidepressant is low. |
| SIGN, 2013,^35^ UK | Individual prescribing for service users in the first episode of psychosis should consider benefits and harms. Minimum effective dose of either FGA or SGA should be used in individuals in the first episode of schizophrenia.  In service users with an acute exacerbation or recurrence of schizophrenia prescribers should consider amisulpride, olanzapine or risperidone as the preferred medications with chlorpromazine and other low-potency FGA providing suitable alternatives. Consideration should be given to previous response to individual AP medications and relative adverse effect profiles. | Following initiation of an AP medication for service users in the first episode of psychosis, the medication should be continued for at least two weeks unless there are significant tolerability issues. Assessment of dose and response should be monitored during the early phase of prescribing. If there is no response to medication after four weeks, despite dose optimization, a change in AP should be considered.  For maintenance treatment, prescribers should consider amisulpride, olanzapine or risperidone as the preferred medications with chlorpromazine and other low-potency FGAs providing suitable alternatives. | For service users with persistent negative symptoms despite adherence to AP medication, consider augmentation with an antidepressant, lamotrigine, or sulpiride. | Individuals who meet criteria for depressive disorder should be treated according to relevant clinical practice guidelines for depression, including the use of antidepressant medication.  SGA should be considered for individuals with schizophrenia which is in remission who have comorbid depressive symptoms. |
| Singapore Ministry of Health, 2011,^36^ Singapore | People newly diagnosed with schizophrenia should be offered oral AP medication. Clinicians must provide information and discuss the benefits and side effect profile of each drug with the patient.  Oral APs should be used as first -line treatment for patients with an acute relapse of schizophrenia. Choice of AP should take into account the patient's previous treatment response, side effect experience, comorbid conditions, compliance history and preference | The recommended optimal oral dose of AP is 300-1,000 mg chlorpromazine equivalents daily for an adequate duration of 4-6 weeks. Treatment should be started at the lower end of the licensed dosage range and slowly titrated upwards.  Combination of APs is not recommended except during transitional periods when patients are being switched from one AP to another, or when used for clozapine augmentation. | NR | Antidepressants should be considered when depressive symptoms emerge during the stable phase of schizophrenia (post -psychotic depression). |
| Schizophrenia PORT, 2010,^30,31^ US | AP medications, other than clozapine and olanzapine, are recommended as first-line treatment for persons with schizophrenia experiencing their first acute positive symptom episode. People with first-episode schizophrenia exhibit increased treatment responsiveness and an increased sensitivity to adverse effects compared with people  with multi-episode schizophrenia. Therefore, AP treatment should be started with doses lower than those recommended for multi-episode patients (first-generation APs: 300–500 mg CPZ equivalents; risperidone and olanzapine: lower half of recommended dosage range for multi-episode patients). An  important exception is with quetiapine, which often requires titration to 500–600 mg/day. The therapeutic efficacy of low-dose aripiprazole or ziprasidone has not been evaluated in people with first-episode schizophrenia | The initial choice of AP medication or the decision to switch to a new AP should be made on the basis of individual preference, prior treatment response, and side effect experience; adherence history; relevant medical history, and risk factors; individual medication side effect profile; and long-term treatment planning. | NR | NR |
| Italian Guidelines, 2008,^42^ Italy | Pharmacologic treatment of first-episode patients is recommended. The choice between typical or atypical APs should consider both the lower incidence of extra-pyramidal symptoms and the higher risk of metabolic side effects because of atypical APs. There is clear evidence of pharmacologic treatments effectiveness to prevent relapse, during the critical period after psychotic onset. The use of AP medications in this phase is recommended, while further studies are needed to investigate the effect of such treatments on long-term prognosis. | NR | NR | NR |
| TMAP, 2008,^28^ US | Treatment of a first-episode schizophrenia includes monotherapy with an SGA AP, such as aripiprazole, olanzapine, quetiapine, risperidone, or ziprasidone. A lower dose of AP drug is usually required for first-episode patients. There was a lack of consensus on inclusion of FGAs as an option for first-episode treatment. | Clinical algorithm:  First line (stage 1): SGA, second line (stage 2): an alternative SGA to what was tried in first line or an FGA, third line (stage 3): clozapine | NR | SSRIs, venlafaxine XR, bupropion SR/XL, duloxetine and mirtazapine are recommended as first-line treatments for depression in schizophrenia |
| NJDMHS, 2005,^32^ US | For first-episode schizophrenia use a low dose of an atypical AP. For a patient with multiple previous trials of medication, select medication according to patient preferences, medication side-effect profiles, history of past response, and cost effectiveness. Give preference to use of an atypical AP if there is no reason to use a conventional agent, because atypical APs have more favorable side effect profiles. | Follow treatment algorithm starting with AP monotherapy for a period of at least four weeks and assess response. In cases of non-response, a new AP monotherapy should be initiated for 4 weeks until a good response without intolerable side effects is achieved. | Atypicals have not been demonstrated to be more effective than conventional agents in treating negative symptoms. While the claim of such benefits continues to be made, the available empirical evidence suggests that the atypical agents are only more effective in reducing secondary negative symptoms. Primary negative symptoms correlate with the deficit syndrome of schizophrenia, but secondary negative symptoms may be due to a variety of causes, including EPS side effects, dysphoria secondary to psychosis or to AP medication, and/or a lack of environmental stimulation | Try to adjust the AP dose first before adding another agent. If the patient is acutely psychotic, increase the dose to treat symptoms. If the patient has akinesia or parkinsonian side effects, decrease the dose.  Clozapine has some antidepressant effects and should be considered if antidepressants cannot be used. Clozapine is the only AP that has an indication for effectiveness in preventing suicide.  If psychotic symptoms are adequately treated and DSM IV criteria for a major depressive episode are met, add an antidepressant. There is limited evidence for the effectiveness of antidepressants in depressed individuals with schizophrenia. |

Abbreviations: AP = antipsychotic; FGA = first-generation antipsychotic; LAI = long-acting injectable; NR = not reported; SGA = second-generation antipsychotic; UK = United Kingdom; US = United States

**Supplemental Table 2. Recommendations on maintenance therapy and for treatment-resistant schizophrenia**

| Guideline, Country | Dose during maintenance treatment | Duration of AP maintenance treatment after a 1st episode | Duration of AP maintenance treatment in multi-episode patients | Choice for treatment resistant schizophrenia | Choice for clozapine-resistant schizophrenia |
| --- | --- | --- | --- | --- | --- |
| APA, 2021,^17^ US | Available doses for APs in the US are provided but specific doses are not outlined for use during maintenance therapy. The guideline notes it is important to assess the ongoing benefits and side effects of treatment that may indicate a need for adjustments to medication doses or changes in medications. The optimal dose of medication is one that provides the best medication benefits yet is tolerable in terms of medication side effects. | NR | NR | Clozapine | Evidence base for other treatments is limited. |
| Florida Medicaid Program, 2020,^18^ US | **Oral AP**  Aripiprazole: 10**–**30 mg/day  Asenapine: 10**–**20 mg/day  Brexpiprazole: 2**–**4 mg/day  Cariprazine: 3**–**6 mg/day  Clozapine: 150**–**800 mg/day  Iloperidone: 12**–**24 mg/day  Lurasidone: 40**–**160 mg/day  Olanzapine: 10**–**20 mg/day  Paliperidone: 3**–**12 mg/day  Quetiapine: 300**–**800 mg/day  Risperidone: 2–8 mg/day  Ziprasidone: 80-160 mg/day | NR | NR | Clozapine  ECT | Use of two APs, ideally with different pharmacological mechanisms (evidence is weak) or FGA. |
| BAP, 2020,^33^ UK | Trial results generally confirm the efficacy of standard dosages of AP medication for relapse prevention but leave the optimal dosage ranges for the available APs undetermined. While a consistent finding has been that a reduced dosage tends to be associated with an increased risk of relapse over time, some advantages with lower dosages have been reported, such as a lower incidence of extrapyramidal side effects. | 2 years | 2 years | Clozapine | If an adequate trial of clozapine monotherapy proves to be of limited efficacy, augmentation strategies may be considered, although few of these drug combinations have a clear rationale and none has a convincing evidence base.  The use of a high-dose or combined AP medication regimen for treatment-refractory schizophrenia should be treated as a limited therapeutic trial, with close monitoring of side effects and therapeutic response. The high dosage should be continued after 3 months only if there is evident clinical benefit that outweighs any risks. |
| Oregon Health Authority, 2019,^19^ UK | A maintenance dose is often lower than an acute treatment dose.  In determining the dose, the urgency for symptom improvement must be weighed against the risk of intolerable side effects. If a patient is willing to accept more potential side effects to experience improvement more quickly, a higher starting dose is acceptable. Alternately, patients who cannot tolerate excessive side effects, or who have no urgency to address their symptoms, may do best on a lower starting dose (10-50 percent of the lowest therapeutic dose). | Consider taper after six to 12 months clinical stability.  Taper medication dose over one to three months (or longer). When proceeding with a medication taper, the patient should have increased contact with their treatment providers. | NR | Clozapine | - Consider augmentation with FGA or SGA dependent upon patient-specific factors  -Maximize non-medication treatments.  -Consult with a psychosis specialist.  -All subsequent medication steps have lower quality of evidence.  -Consider ECT |
| PPA, 2019,^37,38^ Poland | In schizophrenia maintenance therapy the lowest effective dose should be selected. | NR | NR | NR | NR |
| AACP, 2017,^29^ US | LAI administration:  Haloperidol 50mg q4w  Fluphenazine 25 mg q2-3w  Paliperidone palmitate (Sustenna) 156 mg q4w  Paliperidone palmitate (Trinza): q12w 546 mg  Aripiprazole (Maintena) 400 mg q4w  Aripiprazole (Aristada) Lauroxil dose and schedule vary, q4w, q6w, and q8w dosing available  Risperidone LAA “Consta” dose varies based on oral dose conversion, q2w | NR | NR | NR | NR |
| CPA, 2017,^39^ Canada | Following an acute episode of schizophrenia, individuals should be offered maintenance treatment with AP medication at low or moderate regular dosing of around 300 to 400 mg of chlorpromazine equivalents, 4 to 6 mg of risperidone, or other equivalents daily. | ≥18 months | Patients should be offered maintenance treatment and AP medication for 2 and possibly up to 5 years or longer | Clozapine | While the addition of other APs or ECT has perhaps garnered the most attention, and in both cases, there are RCTs available, the consensus of CPA is that, at present, there is insufficient evidence to make any specific recommendation for treatment of clozapine-resistant patients. |
| UNHCR, 2017,^23^ International | Haloperidol: maintenance dose of 4 to 6 mg/day Chlorpromazine: maintenance dose of 75 to 300 mg/day Risperidone: maintenance dose of 4 to 6 mg/day Olanzapine: maintenance dose of 10 mg/day | NR | NR | Clozapine | NR |
| WFSBP, 2012^24^, 2013^25^, 2017,^26^ International | If possible, APs administered during the acute episode should be used for maintenance treatment as well.  Dosages should be titrated at the lowest possible range with the best risk-benefit ratio. Early-episode patients usually need lower dosages than patients with a longer disease course. | Continuous AP maintenance treatment for at least 1 year after the first-episode | Continuous AP maintenance treatment for 2–5 years in case of a recurring course | Clozapine | In cases of clozapine intolerance, a switch to another SGA, preferentially olanzapine or risperidone, should be performed. |
| RANZCP, 2016,^40^Australia and New Zealand | Daily starting and maximum recommended daily doses are provided by AP agent; however, specific doses are not outlined for maintenance treatment.  The guideline does note that if dose reduction is indicated, it should be performed gradually to avoid withdrawal effects and rebound psychoses. | Continuous AP for 2-5 years | In established illness, it is generally considered advisable to continue maintenance treatment with the prescribed AP to which the person responded in the acute episode, as long as the efficacy and benefits outweigh the side effects. However, if the drug is causing adverse effects, then switching to a drug with less potential for causing these side effects should be considered. | Clozapine | Continue clozapine and prescribe adjunctive medication or reinstate the previous regimen that was most effective and well tolerated and prescribe adjunctive medication. |
| NICE, 2014,^34^ UK | During the maintenance period AP medication should be reviewed annually, including observed benefits and any side effects. The choice of drug should be influenced by the same criteria recommended for starting treatment. Consider offering depot /LAI AP medication to people with psychosis or schizophrenia: who would prefer such treatment after an acute episode where avoiding covert non-adherence (either intentional or unintentional) to AP medication is a clinical priority within the treatment plan. | NR | NR | Clozapine | For people with schizophrenia whose illness has not responded adequately to clozapine at an optimized dose, healthcare professionals should review the diagnosis, establish if there have been adherence to medication, and consider other causes of non-response before adding a second AP to augment treatment with clozapine.  An adequate trial of such an augmentation may need to be up to 8–10 weeks. Choose a drug that does not compound the common side effects of clozapine. |
| AFPBN, 2013,^41^ France | It is recommended to continue as maintenance treatment the therapeutic strategy that allowed the reduction of symptoms and the stabilization of the episode (strategy of choice).  In the case of a switch to an oral AP treatment during the acute phase, switching to an LAI formulation as maintenance treatment is recommended as the 1st line strategy. | NR | NR | NR | NR |
| CINP, 2013,^22^ International | Maintenance treatment should be given continuously rather than using an intermittent approach. It is recommended to keep the dose that was effective in the acute phase as long as there are no important side-effects. | At least 1 year | At least 6 years | Clozapine | When clozapine fails, evidence on augmenting clozapine with other treatments is currently scarce. There is some evidence on addition of lamotrigine or topiramate or augmenting with other APs, though evidence is limited on this approach. ECT may be considered as a last resort. |
| SIGN, 2013,^35^ UK | Individuals with schizophrenia, which is in remission, should be offered maintenance treatment with AP medication at low to moderate regular dosing of around 300-400 mg of chlorpromazine, 4-6 mg of risperidone, or their equivalents daily. | At least 18 months | Minimum of 2 years | Clozapine | A trial of clozapine augmentation with a second SGA should be considered for service users whose symptoms have not responded adequately to clozapine alone, despite dose optimization. Treatment should be continued for a minimum of ten weeks.  A trial of clozapine augmentation with lamotrigine may also be considered. |
| Singapore Ministry of Health, 2011,^36^ Singapore | For maintenance therapy, AP dose should be reduced gradually to the lowest possible effective dose, which should not be lower than half of the effective dose during the acute phase. | NR | NR | Clozapine | NR |
| Schizophrenia PORT, 2010,^30,31^ US | The maintenance dosage for first-generation APs should be in the range of 300–600 CPZ equivalents per day. The maintenance dosage for aripiprazole, olanzapine, paliperidone, quetiapine, risperidone, and ziprasidone should be the dose found to be effective for reducing positive psychotic symptoms in the acute phase of treatment. | NR | NR | Clozapine | If a person treated with clozapine has failed to demonstrate an adequate response, then a clozapine level should be obtained to ascertain whether the clozapine level is above 350 ng/ml. If the blood level is less than 350 ng/ml, then the dosage should be increased, to the extent that side effects are tolerated, to achieve a blood level above 350 ng/ml. |
| Italian Guidelines, 2008,^42^ Italy | NR | NR | NR | NR | NR |
| TMAP, 2008,^28^ US | The evidence on maintenance dose is unclear as while the approach of using the lowest effective dose for adequate control of symptoms, these very low doses can lead to relapse in some patients. Therefore, a dose somewhat higher than what would prevent symptoms is best. | Indefinitely | Indefinitely | Clozapine | There is inconsistent evidence that augmentation with SGA, FGA, or ECT has efficacy in treating clozapine resistance. The value of trying a new SGA or FGA that was not tried earlier has not been established. |
| NJDMHS, 2005,^32^ US | Continue acute-phase medication treatment as maintenance on lowest effective dose for adequate duration. | 9-12 months | At least 2-3 years | Clozapine | A specialist consult or review by an outside expert is warranted in cases of clozapine-resistance. ECT can be considered. There is no evidence on the efficacy of treatment with two APs. |

Abbreviations: AP = antipsychotic; ECT = electroconvulsive therapy; FGA = first-generation antipsychotic; LAI = long-acting injectable; NR = not reported; SGA = second-generation antipsychotic; UK = United Kingdom; US = United States
